# Editorial: Neglected tropical diseases: drug targets and potential treatments

**DOI:** 10.3389/fcimb.2026.1927910

**Published:** 2026-09-09

**Authors:** Sanket Kaushik, Biswajit Mishra, Sanjit Kumar, Vijay Kumar Srivastava

**Affiliations:** 1 Amity Institute of Biotechnology, Amity University Rajasthan, Jaipur, Rajasthan, India; 2 Houston Methodist Research Institute, Houston, TX, United States; 3 Guru Ghasidas Vishwavidyalaya, Bilaspur, India; 4 Institute of Science, Nirma University, Ahmedabad, Gujarat, India

**Keywords:** AMR, CRISPR, global burden, infectious disease, neglected tropical disease

The articles in this Research Topic highlight an urgent issue: the global burden of infectious diseases is not diminishing, and the tools available to clinicians, diagnosticians, and public health authorities must evolve faster than the pathogens they target. The global burden of infectious diseases continues to challenge healthcare systems despite substantial advances in diagnostics, therapeutics, and biomedical technologies. Rising antimicrobial resistance, the persistence of neglected tropical diseases, and widening disparities in healthcare access underscore the need for integrated translational research. The studies in this Research Topic collectively highlight emerging innovations spanning molecular diagnostics, green nanotechnology, computational drug discovery, and healthcare quality improvement. Rather than representing isolated advances, these contributions illustrate how multidisciplinary approaches are converging to bolster the prevention, diagnosis, treatment, and resilience of health systems in the face of infectious diseases. This editorial offers a thematic reading of these contributions and considers what they reveal collectively about the direction of translational biomedical science.

The technologies that Zhang et al. review — isothermal amplification platforms, such as LAMP and RPA, CRISPR-based assays, nanomaterial biosensors, and integrated microfluidic systems — represent a genuine architectural shift. Results achievable in under 60 minutes without centralized laboratories are no longer aspirational; they are demonstrable. Platforms capable of detecting resistance-conferring mutations in tuberculosis within 2 hours are already being deployed in the field.

Three of the studies in this Research Topic focus on silver nanoparticles synthesized through biogenic routes — using plant extracts, growth hormones, or agricultural waste as reducing and capping agents. The appeal of green synthesis is not merely aesthetic. Conventional chemical routes for AgNP production involve toxic reagents, generate hazardous byproducts, and have a cost structure poorly suited to low-resource settings. The studies by Sahil et al. (using auxin-class plant hormones), Kim et al. (using radish root filtrate), and Ghodake et al. (using ginger rhizome extract) demonstrate that biogenic routes can yield stable, morphologically well-defined nanoparticles with potent antibacterial activity against clinically significant organisms, including multidrug-resistant strains of *Staphylococcus aureus* and *Escherichia coli*.

The nano-waste management concern raised by Kim et al. operates in the same register. Green synthesis describes the method of production, not the ecological footprint of disposal.

Leishmaniasis remains among the most neglected of the neglected tropical diseases — a group defined less by epidemiological obscurity than by chronic underinvestment. The three contributions addressing it in this Research Topic approach the therapeutic gap from complementary angles, and their juxtaposition is instructive.

The network meta-analysis by Fan et al. provides the most granular comparative efficacy and safety data assembled thus far for the five principal anti-leishmanial agents. The finding that amphotericin B has the highest SUCRA ranking for clinical cure while pentavalent antimony has the greatest mortality risk should prompt a re-examination of first-line treatment protocols in settings where antimony remains the standard of care.

While the Fan et al. meta-analysis maps the existing pharmacological landscape, the computational studies by Yasar et al. and Abdelkrim et al. attempt to expand upon it.

The quality improvement study by Mai et al. addresses a domain that receives insufficient attention in high-impact biomedical literature: the sterile processing infrastructure on which clinical outcomes silently depend.

A review article published by Zhang et al. reports that rapid diagnosis remains a cornerstone of effective infectious disease management. Novel point-of-care molecular platforms based on isothermal amplification, CRISPR technologies, nanobiosensors, and microfluidic systems offer the possibility of delivering accurate results in resource-limited settings while reducing dependence on centralized laboratories. These technologies have the potential to improve antimicrobial stewardship and strengthen disease surveillance.

In conclusion, this Research Topic showcases the breadth of contemporary infectious disease research while emphasizing that scientific advances achieve their greatest impact only when coupled with effective implementation, interdisciplinary collaboration, and supportive public health policy. By bridging fundamental science, translational medicine, and healthcare systems research, the contributions in this Research Topic provide valuable perspectives that will inform future strategies for combating infectious diseases worldwide.

